# Comparison of Surgical Complications Rates Between LigaSure Small Jaw and Clamp-and-Tie Hemostatic Technique in 1,000 Neuro-Monitored Thyroidectomies

**DOI:** 10.3389/fendo.2021.638608

**Published:** 2021-04-07

**Authors:** Cheng-Hsin Liu, Chih-Chun Wang, Che-Wei Wu, Yi-Chu Lin, I-Cheng Lu, Pi-Ying Chang, Ching-Feng Lien, Chien-Chung Wang, Tzer-Zen Hwang, Tzu-Yen Huang, Feng-Yu Chiang

**Affiliations:** ^1^ International Thyroid Surgery Center, Department of Otolaryngology-Head and Neck Surgery, Kaohsiung Medical University Hospital, Faculty of Medicine, College of Medicine, Kaohsiung Medical University, Kaohsiung, Taiwan; ^2^ Department of Otolaryngology-Head and Neck Surgery, Kaohsiung Municipal Siaogang Hospital, Kaohsiung Medical University Hospital, Faculty of Medicine, College of Medicine, Kaohsiung Medical University, Kaohsiung, Taiwan; ^3^ Department of Otolaryngology-Head and Neck Surgery, E-Da Hospital, Kaohsiung, Taiwan; ^4^ School of Medicine, College of Medicine, I-Shou University, Kaohsiung, Taiwan; ^5^ Department of Anesthesiology, Kaohsiung Municipal Siaogang Hospital, Kaohsiung Medical University Hospital, Faculty of Medicine, College of Medicine, Kaohsiung Medical University, Kaohsiung, Taiwan; ^6^ Department of Anesthesiology, Kaohsiung Municipal Ta-Tung Hospital, Kaohsiung Medical University Hospital, Faculty of Medicine, College of Medicine, Kaohsiung Medical University, Kaohsiung, Taiwan; ^7^ Department of Biological Science and Technology, National Yang Ming Chiao Tung University, Hsinchu, Taiwan

**Keywords:** recurrent laryngeal nerve palsy, neuro-monitored thyroid surgery, LigaSure Small Jaw, postoperative hematoma, postoperative hypocalcemia

## Abstract

Over the past decade, the use of neuromonitoring in thyroid surgery has become well established and is increasing accepted across the world. In addition, new developments in energy devices have significantly improved efficacy in achieving hemostasis in thyroid surgery. Few studies focused on the complication rates in energy device-assisted sutureless neuro-monitored thyroidectomy. This study investigates a novel LigaSure Small Jaw (LSJ) technique for sutureless thyroidectomy and compares the surgical complication rates between LSJ and conventional clamp-and-tie technique in one thousand consecutive neuro-monitored thyroidectomy patients. Five hundred patients received sutureless thyroidectomy performed with LSJ (Group L), and 500 patients received surgery performed with conventional clamp-and-tie technique (Group C). Complication rates of postoperative hematoma, hypocalcemia and recurrent laryngeal nerve (RLN) palsy were compared between groups. The overall complication rates of hematoma, hypocalcemia (temporary/ permanent), and RLN (temporary/ permanent) palsy were 0.9%, 24.9% (24.6%/0.3%), and 1.7% (1.5%/0.2%), respectively. Group L and Group C significantly differed in postoperative hematoma rate (0.0% vs. 1.8%, respectively; *p* = 0.0026) and in postoperative hypocalcemia rate (20.1% vs. 30.0%, respectively; *p* = 0.0032). The incidence of RLN palsy did not significantly differ between Group L and Group C (1.38% vs. 2.08%; *p* = 0.2652). The overall surgical complication rates are low in neuro-monitored thyroidectomy. The LSJ is feasible for performing completely sutureless thyroidectomy and obtains superior outcomes of postoperative hematoma and hypocalcemia in comparison with clamp-and-tie hemostatic technique. The novel LSJ technique using double or overlapped sealing is useful for sutureless thyroidectomy. However, surgeons must carefully observe the tissue contraction that may reduce the LSJ-RLN distance and increase the risk of thermal injury during the LSJ activation.

## Introduction

Thyroidectomy is the most common neck endocrine surgery, it’s a high-precision surgical procedure that has several potential complications, including wound infection, seroma or hematoma, laryngeal nerve injury, hypocalcemia, laceration of the trachea or esophagus, chyle fistula, dysphagia, etc. (1). Postoperative hematoma, hypocalcemia and recurrent laryngeal nerve (RLN) palsy are the most common surgical complications of thyroid surgery that may severely impair quality of life and can even be fatal. Therefore, thyroid surgeons must make their best efforts to decrease the potential occurrence of these surgical complications.

Over the past decade, the use of intraoperative neuromonitoring (IONM) techniques in neck endocrine surgeries has gained wide acceptance as an adjunct technique for mapping and identifying the laryngeal nerves, for intermittent or continuous providing functional status on the RLN, for confirming and elucidating mechanisms of RLN injury, for detecting RLN anatomic variations, and for predicting the outcome of vocal cord function in case of cancer invasion or complete/incomplete/recovery loss of signal (2–9), because it adds a new functional dynamic to surgery and empowers surgeons beyond what is available to them through visual information alone. In addition to IONM techniques, new developments in energy-based devices (EBDs) have also significantly improved efficacy in achieving hemostasis in thyroid surgery, which reduces intraoperative blood loss and operation time (10). LigaSure™ Small Jaw (LSJ; Medtronic, Covidien, Colorado, USA), an EBD used in a vessel sealing system, has been widely used and researched in thyroidectomy (11–13). Several studies have reported that, compared to the conventional clamp-and-tie technique, LSJ reduces intraoperative blood loss and has better surgical outcomes in terms of pain and length of hospital stay (14–16). However, the heat generated from EBD is still a major concern for thyroid surgeon when applying this instrument near the RLN. This study investigates the feasibility of performing completely sutureless neuro-monitored thyroidectomy with our novel LSJ technique and compares the surgical complication rates of postoperative hematoma, hypocalcemia and RLN palsy between LSJ and conventional clamp-and-tie hemostatic technique.

## Material and Methods

This retrospective study evaluated 1,000 consecutive patients who had received standardized neuro-monitored thyroidectomy performed by a single surgeon (F-YC) in Kaohsiung Medical University Hospital (17), Taiwan, from October, 2013, to June, 2019. Ethical approval of this study was obtained from the Kaohsiung Medical University Hospital Institutional Review Board (KMUHIRB-E(I)-20190200). The Group C included 500 patients who had received thyroidectomy by conventional clamp-and-tie surgical technique from October, 2013, to October, 2016. In our institution, Ligasure has been routinely used in all thyroid surgery since November 2016. The Group L included 500 patients who had received sutureless thyroidectomy performed by LSJ from November, 2016, to June, 2019.

All thyroidectomies were performed with the application of intermittent intraoperative neuromonitoring (IONM) because of surgeon’s preference (familiarity, price, and accessibility). The electromyographic (EMG) signal was recorded on a nerve integrity monitor (NIM-Response 3.0 System, Metronic Xomed, Jacksonville, FL, USA). Equipment setup and anesthesia were performed according to the standard procedures used by the IONM team at Kaohsiung Medical University Hospital (17). In all patients, vagus nerve and RLN function were routinely evaluated using the standard four-step (V1-R1-R2-V2) IONM procedure (8, 18). In Group C, the branches of major vessels were ligated by clamp-and-tie technique. In Group L, all vessel branches were sealed and cut by LSJ without any clamp-and-tie. The technique of double sealing (two LSJ activations at a single site) or overlapped sealing (two LSJ activations with some tissue overlap) was performed routinely before cutting the large vessels or isthmus of thyroid. (**Supplementary Video 1**) We routinely placed a drain after operation in both groups. Operative time was calculated from start of skin incision to finish of skin closure. In order to present a comparative operative time difference in the groups, the comparison was only performed for patients received TT without Graves’ disease or lateral neck dissection.

In all thyroidectomies performed in this series, preserving parathyroid glands (PGs) in situ was the preferred surgical strategy. PG autotransplantation was only performed in devascularized glands. Serum ionized calcium (iCa) levels were measured in all patients undergoing total thyroidectomy before and 12, 24, 48, and 72 hours after surgery. Normal iCa was defined as mean (± 2SD) preoperative iCa (4.2-5.2 mg/dL). Postoperative hypocalcemia was defined as iCa under 4.2 mg/dL in at least two measurements. Permanent hypocalcemia was considered if a patient with hypocalcemia still required calcium supplements 12 months after surgery. Additionally, vocal cord function was routinely video-recorded before and after surgery. Patients with vocal cord dysfunction were refer to speech therapy and received laryngoscopic examination every month. Permanent RLN palsy was regarded when the vocal cord palsy persisted for more than 6 months. Postoperative hematoma was defined as progressive neck swelling, with or without accompanying respiratory distress, that required emergent surgical intervention (19).

### Statistical Analysis

For analysis of variables, independent t test and Pearson chi-square test were performed using MedCalc for Windows, version 19.1 (MedCalc Software, Ostend, Belgium). A two-tailed P value less than 0.05 was considered statistically significant.

## Results

**Table 1** shows the demographic and clinical characteristics of the patients. Group L and Group C did not significantly differ in age, gender, pathology results, or extent of surgery type. The median (interquartile range) operative time was 99 (82 – 115) minutes in Group L, and was 110 (92 -127) minutes in Group C.

**Table 1:** Demographic and characteristics between groups.

	Total	Group L *n*=500	Group C *n*=500	*p* value
Age (Year)	51.6 ± 13.1	51.7 ± 13.0	51.5 ± 13.2	.6969
Sex(M/F)	182/818	85/415	97/403	.3256
Pathology				.4800
Benign (%)	589(58.9%)	289(57.8%)	300(60.0%)	.9362
TT	378(64.2%)	185(64.0%)	193(64.3%)	
Graves’ disease	23 (2.3%)	12 (2.4%)	11 (2.2%)	
TL	211(35.8%)	104(36.0%)	107(35.7%)	
Malignant (%)	411(41.1%)	211(42.2%)	200(40.0%)	.8361
TT	359(87.3%)	185(87.7%)	174(87.0%)	
TL	52(12.7%)	26(12.3%)	26(13.0%)	
Surgery (TT/TL)	737/263	370/130	367/133	.8295
Operative time* [Median (IQR) minutes]		**99 (82-115)**	**110 (92-127)**	

TT, Total thyroidectomy; TL, Total lobectomy; IQR, Interquartile range.

*The duration from start of skin incision to finish of skin closure in TT cases without Graves’ disease of lateral neck dissection. 336 patients in Group L and 338 patients in Group C were evaluated.

**Table 2** shows the overall complication rates of hematoma, hypocalcemia (temporary/ permanent), and RLN (temporary/ permanent) palsy were 0.9%, 24.9% (24.6%/0.3%), and 1.7% (1.5%/0.2%), respectively.

**Table 2:** Surgical complication rates between groups.

	Total	Group L	Group C	*p* value
Complications				
Postoperative Hematoma *(%*)	9(0.9%)	0(0.0*%*)	9(1.8*%*)	.0026
Postoperative Hypocalcemia^†^ (*%*)	186/746(25.0%)	76/379(20.1*%*)	110/367(30.0*%*)	.0032
Temporary	184(24.7*%*)	76(20.1*%*)	108(29.4*%*)	
Permanent	2(0.3%)	0(0.0*%*)	2(0.6*%*)	
RLN palsy^‡,§^ (*%*)	30/1737(1.72*%*)	12/870(1.38*%*)	18/867(2.08*%*)	.2652
Temporary	26(1.50*%*)	10(1.15*%*)	16(1.85*%*)	
Permanent	4(0.23*%*)	2(0.23*%*)	2(0.23*%*)	

RLN, Recurrent laryngeal nerve.

^†^Incidence of postoperative hypocalcemia is limited to patient with total thyroidectomy. ^‡^No occurrence of bilateral vocal palsy. ^§^Incidence of nerves at risk.

The incidence of postoperative hematoma requiring emergent surgical intervention was significantly lower in Group L compared to Group C (0.0% vs. 1.8%; *p* = 0.0026). The 9 (1.8%) patients with hematoma in Group C were successfully treated without long-term morbidity (**Table 2**).

In the total thyroidectomy patients, postoperative hypocalcemia occurred in 76 cases in Group L (76/379 = 20.1%) and in 110 cases in Group C (110/367 = 30.0%), which was a significant difference between groups (*p* = 0.0032). Two cases in Group C developed permanent hypocalcemia (**Table 2**). All patients with hypocalcemia were continuous monitored and well-controlled with calcium and vitamin D supplementation.

The RLN injury patterns were shown in **Table 3**. Unilateral RLN palsy occurred in 12/870 nerves of Group L and in 18/867 nerves of Group C. The incidence of RLN palsy did not significantly differ between Group L and Group C (1.38% vs. 2.08%; *p* = 0.2652). Among the 12 nerves with postoperative RLN palsy in Group L, four nerves (33.3%) injured by traction and another four nerves (33.3%) injured by mechanical trauma during nerve dissection all showed temporary palsy. The remaining four nerves (33.3%) injured by lateral thermal spread; two nerves showed temporary palsy but the other two nerves showed permanent palsy. Tissue contraction can reduce the safe distance during LSJ activation and can increase the risk of thermal injury (**Supplementary Video 2**). In this video, an adequate safe distance had been reserved before LSJ activation, and the EMG signal of the nerve had not changed after this hemostatic technique.

**Table 3:** Recurrent laryngeal nerve injuries patterns.

	Compression	Thermal	Traction	Sacrificed^†^	Total
Group L	4(33.3*%*)	4(33.3*%*)	4(33.3*%*)	–	12
Transient	4	2	4	–	10
Permanent	–	2	–	–	2
					
Group C	6(33.3*%*)	–	10(55.6*%*)	2(11.1*%*)	18
Transient	6	–	10	–	16
Permanent	–	–	–	2	2

^†^Sacrificed due to nerve encasement by the tumor.

Of the 18 nerves in Group C, 10 nerves (55.6%) injured by traction, and six nerves (33.3%) injured by mechanical trauma during nerve dissection all showed temporary palsy, while the other two nerves (11.1%) were intentionally sacrificed due to nerve encasement by tumor and showed permanent palsy (**Table 3**).

## Discussion

Over the past decade, the use of IONM techniques in neck endocrine surgeries worldwide has progressively risen and with this expansion has come novel research directed at improving IONM procedural and technical factors and developing new monitoring techniques. This study investigates a novel LSJ technique for sutureless thyroidectomy and compares the surgical complication rates between LSJ and conventional clamp-and-tie technique in 1000 consecutive neuro-monitored thyroidectomy patients. The result show that the overall complication rates is low in neuro-monitored thyroidectomy, and the LSJ is feasible for performing completely sutureless thyroidectomy and obtains superior outcomes of postoperative hematoma and hypocalcemia in comparison with clamp-and-tie hemostatic technique (**Table 2**). The novel LSJ technique using double or overlapped sealing before cutting the large vessels or isthmus of thyroid is useful for sutureless thyroidectomy (**Supplementary Video 1**). However, surgeons must carefully observe the tissue contraction that may reduce the LSJ-RLN distance and increase the risk of thermal injury during the LSJ activation (**Supplementary Video 2**).

Postoperative hematoma is a rare but potentially life-threatening complication that requires emergent reoperation to prevent external compression of the airway (20–22). In Weiss et al., a review of 150,012 thyroid surgery patients demonstrated a 1.34% postoperative hematoma rate and a 0.32% mortality rate (23). Several studies have reported that, LSJ achieves hemostasis in thyroidectomy more quickly and more effectively compared to the conventional clamp-and-tie technique; therefore, it reduces overall operating time and intraoperative blood loss. In our study, the median operative time is 11 minutes shorter in Group L than in Group C, which may be contributed to the higher efficiency of bleeding control when using LSJ. However, the occurrence of post-operative hematoma does not significantly differ between LSJ and the conventional technique in some researches (16, 24, 25). In the current study, the extent of surgery and gender showed no significant difference between group, and we routinely placed a drain after operation in both groups. The hematoma rate of 0.9% in all 1000 patients was slightly lower than the rate of approximately 1.3% in large scale studies (23, 26) that were not grouped for hemostasis techniques. In current study, nine cases (1.8%) encountered postoperative hematoma and required emergent surgical intervention in Group C. In contrast, no cases of postoperative hematoma occurred in Group L. In a large retrospective cohort study evaluated 10,903 patients receiving thyroid surgery, 2.42% of patients in conventional hemostasis group and 1.25% of patients in vessel-sealant devices group had postoperative hematoma (27). It might be supposed the critical hydrostatic pressure that the conventional tie could withstand is varied, resulting in a certain probability of postoperative hematoma. While using LSJ, a correct hemostasis procedure of first LSJ activation as the official instruction manual for users is essential in every hemostasis step. Furthermore, the following implementation hemostasis steps we routinely performed like double sealing or overlapped sealing before cutting the large vessels or isthmus of thyroid, which were suggested in the article, will at least produce a hemostasis effect not lower than a single activation. This novel LSJ hemostatic technique has not been reported in the literature (**Supplementary Video 1**). We surmise that, by improving effectiveness in sealing large vessels, double or overlapped sealing can substantially reduce the incidence of postoperative bleeding when LSJ is used for a sutureless thyroidectomy.

Postoperative hypocalcemia is the most common complication after total thyroidectomy. The reported rates of temporary and permanent hypocalcemia in thyroid cancer surgery are as high as 68% and 25%, respectively (28). Postoperative hypocalcemia is also a common cause of hospital readmission (22), and all patients with symptomatic hypocalcemia require intravenous or oral administration of calcium supplements (29). Whether LSJ hemostatic technique reduces the rate of postoperative hypocalcemia is still controversial in the literature. Kuboki et al. reported a significantly lower rate of temporary hypocalcemia in an LSJ group compared to a conventional group; however, the permanent hypocalcemia rate did not significantly differ between groups (24). Chiang et al. also demonstrated a significantly lower rate of postoperative hypocalcemia in an LSJ group compared to a conventional group (30). In another study, however, Pergel et al. reported that the rate of laboratory hypocalcemia was significantly higher in an LSJ group compared to a conventional group whereas the rate of symptomatic hypocalcemia did not significantly differ (31). In this study, the patients undergoing total thyroidectomy, hypocalcemia rates were significantly lower in Group L (20.1%) compared to Group C (30.0%). In order to reserve space for tightening the tie in conventional technique, the position of the tie might be more proximal than the position where Ligasure can be activated, even in its upstream branch. Thyroid gland might also require more retraction in conventional technique, which in turn affects the blood flow of the PG. We hypothesize that, by providing more effective and individualized cauterization of tertiary branches of vessels on the thyroid capsule, LSJ achieves better preservation of the blood supply to the PG.

All patients in this study underwent IONM in accordance with the standard procedure at our institution, which included pre- and post-operative video-recording of vocal cord function and intraoperative four-step (V1-R1-R2-V2) IONM procedure. Therefore, we can determine the actual causes of RLN injury (2, 18). Although the postoperative RLN palsy rate did not significantly differ between Group L and Group C, the two groups had very different patterns of nerve injury, particularly in thermal injury. In Group C, clamp-and-tie technique was performed for hemostasis near the RLN, and no thermal injuries occurred. In Group L, however, four nerves had thermal injury caused by lateral thermal spread of LSJ. Of the four nerves, two nerves had complete loss of electromyography (EMG) signal resulting in permanent vocal cord palsy; in the other two nerves, incomplete loss of EMG signal resulted in temporary cord palsy. Although several studies have also reported that RLN palsy rates did not significantly differ between LSJ and conventional clamp-and-tie technique (16, 25), the potential risk of thermal injury caused by LSJ is rarely emphasized. This study highlights the risk of thermal injury caused by lateral heat spread when LSJ is used near RLN. Since thermal injury to the RLN is difficult to be detected visually and the risk of permanent palsy is high after its occurrence. Therefore, LSJ must be used with extreme caution near the RLN. Our previous animal study showed no occurrence of adverse EMG events when LSJ was activated at a distance of 2 to 5 mm from the RLN, and the study concluded that, the safe distance for LSJ activation is 2mm base on EMG functional assessment (13). However, these results should be applied cautiously in clinical practice because tissue contraction can reduce the safe distance during LSJ activation and can increase the risk of thermal injury (**Supplementary Video 2**). Therefore, we recommend that the RLN should be clearly visualized and a safe distance from the nerve should be ensured more than 2mm before LSJ activation, and a standardized IONM procedure is also recommended that the surgeon can facilitate this step more efficiently and precisely.

## Limitation

There are still some limitations regarding this study. This is not a prospective or randomized study, and the surgical procedures were not performed in the same time period. Surgical outcomes may improve with the experience of the operator during the second period. However, all surgical procedures considered in this study were performed by a single surgeon with over 30 years of experience in thyroid surgery, which may decrease the influence of these factors (32). However, there are still differences among surgeons in terms of experience, learning background, and familiarity with new devices. The more effective use of instruments is positive for the surgical outcome.

External branch of superior laryngeal nerve (EBSLN) related dysphonia and postoperative dysphagia are also important surgical complications that were not discussed in this article, these important issues were believed requiring more postoperative subjective and objective voice and swallowing assessments, and should be listed as the future research direction. In addition, we did not compare the factors of different nodule size/weight of the thyroid that are also biases and may potentially influence the risk of major complications.

Parathyroid hormone (PTH) became the gold standard in the diagnosis of postoperative hypoparathyroidism in recent years (33), and has not been performed in all cases in this study. However, the same iCa level evaluation method was used between the groups, which was still comparable.

In this study, though all patients in the Group L can be performed suturelessly under LSJ hemostasis, however, for the tumor margin may be inadequate near the nerves (RLN, EBSLN or vagus nerve), conventional clamp-and-tie should be still considered for the oncological safety.

## Conclusions

The overall complication rates are low in neuro-monitored thyroidectomy. The LSJ is feasible for performing completely sutureless thyroidectomy and obtains superior outcomes of postoperative hematoma and hypocalcemia in comparison with clamp-and-tie hemostatic technique. Since LSJ allows double or overlapped sealing of the targeted tissue, it can improve efficiency in vessel sealing, and substantially reduce the incidence of postoperative bleeding. Additionally, LSJ provides more effective and individualized cauterization of tertiary branches of vessels on the thyroid capsule and achieves better preservation of blood supply to the PG. However, surgeons must carefully consider the potential risk of RLN injury caused by lateral thermal spread from the LSJ. Notably, tissue contraction caused by LSJ activation near the RLN reduces the safe distance and increases the risk of thermal injury.

## Data Availability Statement

The original contributions presented in the study are included in the article/**Supplementary Material**. Further inquiries can be directed to the corresponding authors.

## Ethics Statement

The studies involving human participants were reviewed and approved by Kaohsiung Medical University Hospital Institutional Review Board (KMUHIRB-E(I)-20190200). The ethics committee waived the requirement of written informed consent for participation.

## Author Contributions

FY-C, CW-W, and TY-H conceived and designed the study. Administrative support was obtained by IC-L and PY-C. Provision of study materials or patients by ChihC-W, CF-L, ChieC-W, and TZ-H. CH-L and YC-L had collected and assembled the data. Data analysis and interpretation was done by CH-L, FY-C, and TY-H. All authors contributed to the article and approved the submitted version.

## Funding

This report was supported by grants from the Kaohsiung Medical University Hospital (KMUH 107-7R50, KMUH 108-8M48, KMUH SA10807c), Taiwan, and Ministry of Science and Technology (MOST 108-2628-B-037-006, MOST 109-2628-B-037-014), Taiwan.

## Conflict of Interest

The authors declare that the research was conducted in the absence of any commercial or financial relationships that could be construed as a potential conflict of interest.

## Supplementary Material

Supplementary Video 1 The LSJ can be used with double sealing or overlapped sealing before cutting the large vessels or isthmus of thyroid.

Supplementary Video 2 Tissue contraction can reduce the safe distance during LSJ activation and can increase the risk of thermal injury.
