# Supplementary material for: Comparison of Surgical Complications Rates Between LigaSure Small Jaw and Clamp-and-Tie Hemostatic Technique in 1,000 Neuro-Monitored Thyroidectomies
Source: Front Endocrinol (Lausanne). 2021 Apr 7;12:638608. doi: 10.3389/fendo.2021.638608 (PMC8058413; doi:10.3389/fendo.2021.638608)
Supplement: Supplementary file 3 [file DataSheet_1.docx]

**Legends for Videos**

**Video 1**. The LSJ can be used with double sealing or overlapped sealing before cutting the large vessels or isthmus of thyroid.

**Video 2**. Tissue contraction can reduce the safe distance during LSJ activation and can increase the risk of thermal injury.
